# Sponge-Microbe Associations Survive High Nutrients and Temperatures

**DOI:** 10.1371/journal.pone.0052220

**Published:** 2012-12-20

**Authors:** Rachel Simister, Michael W. Taylor, Peter Tsai, Nicole Webster

**Affiliations:** 1 Centre for Microbial Innovation, The University of Auckland, Auckland, New Zealand; 2 Bioinformatics Institute, School of Biological Sciences, The University of Auckland, Auckland, New Zealand; 3 Australian Institute of Marine Science, Townsville Mail Centre, Qld 4810, Australia; Victoria University Wellington, New Zealand

## Abstract

Coral reefs are under considerable pressure from global stressors such as elevated sea surface temperature and ocean acidification, as well as local factors including eutrophication and poor water quality. Marine sponges are diverse, abundant and ecologically important components of coral reefs in both coastal and offshore environments. Due to their exceptionally high filtration rates, sponges also form a crucial coupling point between benthic and pelagic habitats. Sponges harbor extensive microbial communities, with many microbial phylotypes found exclusively in sponges and thought to contribute to the health and survival of their hosts. Manipulative experiments were undertaken to ascertain the impact of elevated nutrients and seawater temperature on health and microbial community dynamics in the Great Barrier Reef sponge *Rhopaloeides odorabile*. *R. odorabile* exposed to elevated nutrient levels including 10 µmol/L total nitrogen at 31°C appeared visually similar to those maintained under ambient seawater conditions after 7 days. The symbiotic microbial community, analyzed by 16S rRNA gene pyrotag sequencing, was highly conserved for the duration of the experiment at both phylum and operational taxonomic unit (OTU) (97% sequence similarity) levels with 19 bacterial phyla and 1743 OTUs identified across all samples. Additionally, elevated nutrients and temperatures did not alter the archaeal associations in *R. odorabile*, with sequencing of 16S rRNA gene libraries revealing similar *Thaumarchaeota* diversity and denaturing gradient gel electrophoresis (DGGE) revealing consistent *amoA* gene patterns, across all experimental treatments. A conserved eukaryotic community was also identified across all nutrient and temperature treatments by DGGE. The highly stable microbial associations indicate that *R. odorabile* symbionts are capable of withstanding short-term exposure to elevated nutrient concentrations and sub-lethal temperatures.

## Introduction

The Great Barrier Reef (GBR) hosts high biodiversity and is the world’s largest coral reef ecosystem. At almost 2000 km long it was declared a World Heritage Area in 1981 [1]. Degradation of coastal marine ecosystems is occurring globally due to over-fishing, declining water quality and climate change [2], [3]. Despite the GBR’s protected status it is still exposed to anthropogenic and environmental pressures, making degradation due to terrestrial run-off the focus of intense management efforts [4]. Twenty-six major catchments, in which a wide range of industrial and agricultural activities take place, border the GBR [5]. Fertilisers used in cattle grazing, sugarcane production and horticulture can flow into the marine environment [6], with 80% of the total anthropogenic dissolved inorganic nitrogen (DIN) introduced into the GBR ecosystem thought to come from fertilisers (Table 1) [2], [7]. Moreover, nitrogen and phosphorus loads have increased by factors of approximately 6 and 9, respectively, since European settlement ca.1830 [8]. Catchment areas in the GBR are characterized by distinct wet/dry seasonal rainfall and are subject to intense cyclonic rainfall over periods of days to a few weeks [9]. River discharge of nutrients into the GBR therefore occurs almost entirely in large pulse events or flood plumes [6], [10] which generally affect reefs within 20 km of the coast (∼27% of all reefs). The resulting elevated nutrient levels can be 2–100 times higher than ambient [6], [10], [11] but are relatively short-lived, detectable for only 3–14 days after flood plume events [12]. Cyclonic events are increasing in frequency and intensity, with the most recent on the GBR (in January 2011) delivering extremely high levels of nutrients from agricultural and urban catchments to the reef environment [13]–[15]. Nutrient levels in ambient (non-flood) conditions are presented in Table 2.

**Table 1:** Total yearly inputs of anthropogenic nutrient loads into the GBR, ^1^ [2], ^2^ [121].

Anthropogenic nutrient loads^1,2^	Tonnes/year
Total nitrogen (TN)	80000
Dissolved inorganic nitrogen (DIN)	11000
Dissolved organic nitrogen (DON)	6900
Particulate nitrogen (PN)	52000
Total phosphorus (TP)	16000
Dissolved inorganic phosphorus (DIP)	800
Dissolved organic phosphorus (DOP)	470
Particulate phosphorus (PP)	13000

**Table 2:** The level of nutrients in ambient (non flood) waters, reported from Pelorus Island from 2005–2011 ^1^ [4].

Nutrient	NH_4_	NO_3_	DON	PN	DOP	PP	DOC	POC
**Ambient wet season^1^**	0.13	0.06	5.64	1.08	0.14	0.1	70.66	9.44
**Ambient dry season^1^**	0.04	0.03	5.49	0.74	0.17	0.06	59.25	6.93

Parameters are in µM for dissolved inorganic nutrients (NH_4_, NO_3_), dissolved organic nitrogen, phosphorus and carbon (DON, DOP, DOC) and particulate nitrogen, phosphorus and organic carbon (PN, PP, POC).

Marine sponges are important components of coastal and offshore coral reefs, exhibiting high diversity, high biomass and the ability to influence both benthic and pelagic processes [16]. Sponges also harbour extensive microbial communities which can comprise up to 35% of sponge tissue volume and include bacteria, archaea and eukarya [17], [18]. To date, 32 bacterial phyla and candidate phyla have been reported from sponges [19], [20], with some phylotypes appearing to occur exclusively in sponges and not in the surrounding environment i.e. so-called sponge-specific clusters (SCs) or sponge- and coral-specific clusters (SCCs) [17], [21]–[23]. In areas such as coral reefs, where dissolved nutrients and particulate organic matter are scarce, sponges may experience nitrogen limitation and symbiotic microorganisms are thought to contribute to nitrogen cycling within the host. Both autotrophic (such as *Cyanobacteria*) and heterotrophic symbionts may contribute to the nitrogen budget of sponges by fixing atmospheric nitrogen [24]–[26]. In low nutrient waters symbionts are thought to benefit by recycling nitrogenous waste excreted from the sponge host [18]. Ammonia-oxidising bacteria (AOB) of the genera *Nitrosospira* and *Nitrosococcus* [27] and the ammonia-oxidising archaea (AOA), such as “*Candidatus* Cenarchaeum symbiosum” [28], [29], which convert ammonia to nitrite, have all been identified in sponges. Nitrite-oxidising bacteria (NOB) such as *Nitrospina* and *Nitrospira* have also been detected in many sponge species [21], [30]–[33], as have denitrification and anaerobic ammonia oxidation (anammox) processes [31], [34]. Symbiosis between nitrogen-transforming microbes and sponges influences not only the ecology of the host but also the wider reef ecosystem (reviewed by [35]).

Elevated nutrient levels have been highlighted as a cause of coral reef decline, with some studies reporting an increase in the severity of coral diseases such as aspergillosis and yellow blotch [36]–[42]. Additionally, both resilience [43], [44] and sensitivity of coral-microbial associations due to elevated nutrient levels have been reported [45]–[47]. Despite the importance of sponge nitrogen cycling to coral reef ecosystems [35], very little research has addressed the sensitivity of sponge–microbial partnerships to nutrient enrichment, with the effects of eutrophication more widely reported for free-living microbial communities. In general, as the availability of nitrogen and phosphorus increases, phytoplankton and bacterial production increases which leads to a higher biological oxygen demand and increases the sedimentation rate of particulate matter [48]. In the early stages of nutrient loading within Chesapeake Bay [49], bacterioplankton communities remain dominated by SAR11, SAR86 and picocyanobacteria, however as anoxic conditions develop the bacterial community shifts to anaerobic members of the *Firmicutes, Bacteroidetes* and sulphur-oxidising *Gammaproteobacteria*. Another effect commonly observed in nutrient-rich environments is an increased abundance of prokaryotic cells; in natural seawater amendments (e.g. the addition of nutrient-rich deep waters to nutrient-depleted surface waters) an increase in the abundance of taxa such as SAR11 and marine *Actinobacteria* was reported [50]–[52]. Several studies have also addressed the effect of nutrient addition on bacterial community structure in the marine environment [53]–[57], however results have been variable due to different experimental methodologies and the high spatial and temporal variability of free-living marine communities [48]. Despite the variable microbial responses to experimental nutrient amendments, few microbial communities have been shown to be resistant to change after environmental disturbance.

Here we analyzed how the microbial community of the Great Barrier Reef sponge *Rhopaloeides odorabile* responded to experimental nutrient exposures under ambient and elevated seawater temperature. While the interactive effects of multiple stressors have previously been explored in microbial biofilms [58], coral larvae [59], foraminifera [60], coral pathogens [41] and adult corals [61], the impact of combined anthropogenic stressors on marine sponges was unknown. Elevated seawater temperature has previously been shown to cause a shift in the dominant microbial community on marine sponges as well as a decline in sponge health [62]–[64], with some mass mortality events concomitant with anomalies in sea surface temperature [65], [66]. Previous experiments have demonstrated that adult *R. odorabile* exhibit necrosis and a loss of microbial symbionts within 72 h at 33°C [67], with subsequent experiments confirming a narrow thermal threshold for the host and symbiont community between 31–32°C [68], [69]. Here we investigated the combined effects of water quality and elevated seawater temperature by exposing sponges to a range of elevated nutrient levels under ambient (27°C) and sub-lethal (31°C) seawater temperatures.

## Materials and Methods

### Sample Collection and Experimental Design

18 *R. odorabile* individuals were collected by SCUBA from Pelorus Island on the Great Barrier Reef (GBR), Australia (18° 32.710′ S, 146° 29.273′ E) in July 2010. All necessary permits were obtained from the Great Barrier Reef Marine Park Authority for all of the described experimental studies. Donor sponges were cut into a total of 100 clones (each approximately 15 cm^3^) and randomly transferred to plastic racks which were secured with weights to the benthos [70]. The sponge clones were allowed to heal on the reef for 12 weeks before collection and transportation to an indoor aquarium at the Orpheus Island Research Station on the GBR (18°36.5′ S, 146°29.4′ E). Previous research has shown that there is little variability of the microbial communities between replicate clones that come from different donor individuals [71]. The experimental design incorporated three nutrient levels, ambient (unamended seawater), low and medium, and 2 temperatures (27 and 31°C) in 3 replicate 30 L flow-through (400 ml/min) aquaria per nutrient/temperature exposure, each holding 7 sponge clones. All tanks were illuminated using fluorescent tubes under a 12∶12 h diurnal cycle at 80 µmol quanta m^_2^ s^_1^ to reflect light intensity at 15 m on the reef. The experiment was conducted by randomly sampling one clone from each replicate tank, per treatment, at time points* = *0, 1, 5, 7 and 12 days. Treatments were maintained for the first 7 days, then all nutrient dosing was stopped and temperatures were returned to 27°C for the final 5 days of the experiment, serving as a recovery period. Samples were snap-frozen in liquid nitrogen immediately after collection, and maintained at –80°C before DNA extraction and analysis.

### Nutrient Concentrations and Calculations

This study used three nutrient levels: ambient (unamended seawater), low and medium. Stock solutions of Thrive® water-soluble plant fertiliser (NPK; 27∶5.5∶9 and trace elements) were continuously delivered by peristaltic pump (1 ml/min) into 30 L aquaria to final concentrations of (1) total inorganic nitrogen - ambient 1.3 µmol/L, low 1.9 µmol/L and medium 4.7 µmol/L, and (2) total nitrogen - ambient 6.5 µmol/L, low 7.1 µmol/L and medium 10 µmol/L (Table S1a). Nutrient levels were monitored throughout the experiment to ensure treatment levels were maintained (Table S1a). Seawater samples for analysis of dissolved nutrients (DIN, TDN and DOC) were hand-filtered through a 0.45 µm filter cartridge (Sartorius MiniSart) into acid-washed screw-cap plastic test tubes and stored frozen until later analysis. Samples for DOC analysis were filtered, acidified with 100 µL of HCl and stored frozen until analysis. Seawater samples for determination of particulate nutrients were collected by vacuum filtration on pre-combusted glass-fibre filters (Whatman GF/F). Filters were wrapped in pre-combusted aluminum foil envelopes and frozen until analysis. Dissolved and particulate nutrient levels (ammonium, nitrite, nitrate, phosphate, DOC, PN, POC) were analysed by the water quality laboratory at the Australian Institute of Marine Science (AIMS, Townsville) (Table S1a). Concentrations of dissolved inorganic nutrients and total dissolved nutrients were determined using a Bran and Luebbe AA3 segmented flow analyser using methods described by [72]. Concentrations of dissolved organic nutrients were determined by subtraction of the respective dissolved inorganic components (following UV irradiation of the samples to oxidise organic matter) from the levels of total dissolved nutrients.

### DNA Extraction

All tissue samples were processed using an approach previously optimized for marine sponges [73]. Briefly, tissue samples were homogenised using lysing matrix E tubes (MPBio) in combination with a Mini-Beadbeater (Biospec Products, Bartleville, OK, USA). For DNA extraction, a Qiagen AllPrep DNA/RNA Mini kit (Cat. #80204) was used according to the manufacturer’s instructions. Purity and quantity of DNA were assessed using a NanoDrop 1000 spectrophotometer (Thermo Scientific) and gel electrophoresis of a 5 µL aliquot on a 1% agarose gel containing 0.5 µg ml^−1^ ethidium bromide. DNA was extracted from seawater filters by addition of 200 µL lysozyme (10 mg/ml), incubation at 37°C for 45 min, addition of 200 µL of proteinase K (0.2 mg/ml) in 1% SDS and incubation at 55°C for 1 h. Lysates were recovered into fresh Eppendorf tubes and nucleic acids extracted using the Qiagen AllPrep DNA/RNA Mini kit (Cat. #80204).

### Denaturing Gradient Gel Electrophoresis (DGGE)

#### 16S rRNA gene – *Bacteria*

The 16S rRNA genes from each sponge clone and seawater sample were amplified by PCR with primers 1055f: 5′-ATGGCTGTCGTCAGCT-3′ and 1392r: 5′-ACGGGCGGTGTGTAC-3′ [74]. The reverse primer was modified to incorporate a 40 bp GC clamp [75]. Cycling conditions were: 3 min at 95°C, followed by 30 cycles of 1 min at 94°C, 1 min at 54°C, 3 min at 72°C, with a final extension of 7 min at 72°C.

#### *amoA* gene from Ammonia-Oxidizing Archaea (AOA)

The *amoA* gene from ammonia-oxidizing archaea (AOA), which encodes for the catalytic subunit of ammonia monooxygenase [76], was targeted by amplifying a ∼635 bp fragment of the *amoA* gene using primers Arch-amoAF 5′-STAATGGTCTGGCTTAGACG-3′ and Arch-amoAR 5′-GCGGCCATCCATCTGTATGT-3′ [77]. The reverse primer was modified to incorporate a 40 bp GC clamp [75]. Cycling conditions were: 5 min at 95°C, followed by 30 cycles of 45 s at 95°C, 55 s at 55°C, 1 min at 72°C followed by a final extension of 5 min at 72°C. Amplification of ammonia-oxidizing bacteria (AOB) produced fragments of many different sizes so these data were excluded from further analysis.

#### 18S rRNA gene – *Eukarya*

Changes in the eukaryotic microbial community in response to nutrient and temperature treatment were assessed using a eukaryote-specific primer set for the 18S rRNA gene. A subset of samples; Day 0 (control), Day 7 (ambient, low and medium nutrient exposures at both 27 and 31°C), with three replicates from each treatment, was screened by DGGE. The 18S rRNA gene was amplified by PCR with eukaryote-specific primers NS1f: 5′-GTA GTC ATA TGC TTG TCT C-3′ and NS2r: 5′- GGC TGC TGG CAC CAG ACT TGC-3′, [78]. The reverse primer was modified to incorporate a 40 bp GC clamp [75]. Cycling conditions were: 3 min at 95°C, followed by 30 cycles of 30 s at 95°C, 30 s at 55°C, 1 min at 72°C followed by a final extension of 7 min at 72°C.

Products from all PCR reactions were applied to 8% w/v polyacrylamide (37.5∶1) gels containing denaturing gradients made from formamide and urea. 16S rRNA-bacterial and *amoA*-gene gels contained a gradient of 50–70%, while 18S rRNA gels had a 30–70% gradient. Gels were electrophoresed at 60°C for 17 h in 1×TAE (Tris-acetic acid EDTA) buffer at 65 V using the Ingeny D-Code system. Gels were stained with 1×Sybr Gold for 30 min, visualized under UV illumination and photographed (Quantity One; Bio-Rad, Gladesville, New South Wales, Australia).

### Multidimensional Scaling (MDS)

Banding patterns from DGGE were transformed into presence/absence matrices and imported into PRIMER 6 (PRIMER-E, 2006, Ltd). MDS plots were created based on Bray-Curtis similarities, with 10000 iterations of bootstrapping. Hierarchical clustering of similarities was performed using the CLUSTER method and this information was superimposed onto the plots to create contours designating thresholds of similarity.

### Amplification, Cloning, and Sequencing of 16S rRNA Genes from *Archaea*

Archaeal diversity in Day 7 ambient nutrient exposures was compared to Day 7 medium nutrient exposures, by clone library analysis. Two clone libraries were created (one for ambient and one for medium nutrient exposures) by combining all replicate sponges (A, B, C) and temperatures (27 and 31°C) within each of the ambient (n = 6) and medium (n = 6) nutrient treatments from Day 7. A portion of the archaeal 16S rRNA gene was amplified using the primers 21F 5′ TTCCGGTTGATCCYGCCGGA-3′ and 958R 5′TCCGGCGTTGAMTCCAATT-3′ [79]. Cycling conditions were 94°C for 1.5 min, 30 cycles of 94°C for 1 min, 54°C for 1.5 min, and 72°C for 2 min, and a final extension of 5 min at 72°C. Cloning was performed using the P-GemT Easy vector kit (Promega, Inc, Madison WI, USA) according to the manufacturer’s instructions. Clones containing the correct-sized insert were analyzed using amplified ribosomal DNA restriction analysis (ARDRA) [80], [81]. Restriction enzymes *Hha1* and *Hae* III were used to analyze the diversity of archaeal 16S rRNA genes in each clone library. Digests were performed using 5 µL of PCR template with 1 µL of the respective enzyme and 3 µL of reaction buffer for 3 h at 37°C, followed by 20 min at 80°C to halt the reaction. Digested products were visualised on a 2% agarose gel to obtain ARDRA profiles. One to two clones representing each ARDRA banding pattern were selected for sequencing, which was performed on a capillary sequencer (Macrogen Inc, Seoul, South Korea).

#### Phylogenetic analysis of archaea

Archaeal 16S rRNA gene sequences were compared to available databases using the Basic Local Alignment Search Tool (BLAST) [82] to determine approximate phylogenetic affiliations. Chimeric sequences were identified using UCHIME [83] implemented in the program MOTHUR [84]. Sequences were aligned using the SINA Web Aligner [85] and then imported into the ARB programme package for manual editing using the SILVA database [22]. All subsequent phylogenetic analyses were performed in ARB. Maximum likelihood algorithms were used to calculate a phylogenetic tree, with maximum parsimony-based bootstraps (1000 resamplings) also calculated to assess the stability of observed branching patterns.

### 454 Pyrosequencing

A subset of samples (day 0 control and day 7 samples from all treatments) was screened by 454 pyrotag sequencing. The 16S rRNA *Bacteria*-specific sequences (targeting the V4–V5 region) were 454MID_533F (GTGCCAGCAGCYGCGGTMA) and 454_907R (CCGTCAATTMMYTTGAGTTT). Amplification primers were designed with FLX Titanium adapters. Forward primers contained the A adapter (CCA TCT CAT CCC TGC GTG TCT CCG AC) and the reverse primer contained the B (CCT ATC CCC TGT GTG CCT TGG CAG TC). A multiplex identifier (MID) was added to each of the forward 16S primer sequences (Roche Applied Sciences). Touchdown PCR conditions were as follows: 3 min at 94°C followed by 20 cycles of 30 s at 94°C, 30 s at 60°C (−0.5°C per cycle), 45 s at 72°C. This was followed by a further 10 cycles of 30 s at 94°C, 30 s at 50°C, 45 s at 72°C, with a final extension of 10 min at 72°C. For each sample PCR products were pooled from multiple reactions (100 µL total) and purified using AMPure XPbeads (Agencourt, Beckman Coulter, USA). Amplicon quality was checked on an Agilent Bioanalyzer 2100 DNA 1000 chip (Agilent Technologies). The number of molecules for each sample was calculated using size (bp) and concentration (ng/mL) data from the Qubit Quant-iT™ DNA high-sensitivity assay kit and a Qubit® fluorometer (Invitrogen) according to the manufacturer’s instructions. Pyrosequencing was performed using a 454/Roche GS Junior instrument (Roche, NJ, USA) at the School of Biological Sciences, University of Auckland, under the auspices of New Zealand Genomics Limited.

### Processing of Raw Sequence Data

Sequences were processed using a combination of Mothur and custom PERL scripts [84]. Pyrosequencing flowgrams were filtered and denoised using the Mothur implementation of AmpliconNoise [86]. Sequences were removed from the analysis if they were <200 bp, contained ambiguous characters, had homopolymers longer than 8 bp, more than one MID mismatch, or more than two mismatches to the reverse primer sequence. Unique sequences were identified with Mothur, aligned against a SILVA alignment (available at http://www.mothur.org/wiki/Silva_reference_alignment). Sequences were chimera checked using UCHIME [83], then grouped into 97% operational taxonomic units (OTUs) based on uncorrected pairwise distance matrices. A representative sequence (defined in Mothur as the sequence with the minimum distance to the other sequences in the OTU) of each OTU was used for the taxonomic assignment using custom PERL scripts, similar to a previously used approach [19], [87]. For each tag sequence, a BLAST search [82] was performed against a manually modified SILVA database [22]. A Smith-Waterman algorithm was used to create pairwise global alignments between the 10 best hits against a tag sequence. For assignment the most similar sequence to the tag sequence (or multiple sequences if within a range of 0.1% sequence divergence) was used. Sequence similarity thresholds of 75%, 80%, 85%, 90% and 95% were applied for assignment at phylum, class, order, family and genus level, respectively. In cases where the taxonomy of the most similar sequences was inconsistent, a majority rule was applied and the tag was only assigned if at least 60% of all reference sequences shared the same taxonomic annotation at the respective taxonomic level. All previously published, sponge-derived sequences in the SILVA reference database were labelled as such [22] and it was noted when a tag sequence was assigned to a sponge-specific (SC) and/or sponge coral-specific sequence cluster (SCC). For assignment to an SC and/or SCC cluster a 75% sequence similarity threshold was applied.

### Determining the Magnitude of Changes in Bacterial Community Structure

The magnitude of change in bacterial community structure was calculated for the 50 OTUs with the largest fold-change (positive or negative) by first normalising the number of reads per OTU, per sample and the values from three replicates were averaged. Treatment Day 7 values were then divided by Day 0 control values. Fold changes were subsequently Log (base 2)-transformed, so that positive (increases in relative abundance) and negative values (decreases) were weighted equally. Log (base 2)-transformation also means that the mapping space is equal and that positive and negative fold changes are comparable. Values for all samples were then ranked and the top 50 OTUs with the largest fold changes (be that positive or negative) were chosen for further analysis. Data was visualized as heatmaps using JColorGrid [88].

## Results

**Figure 1:**
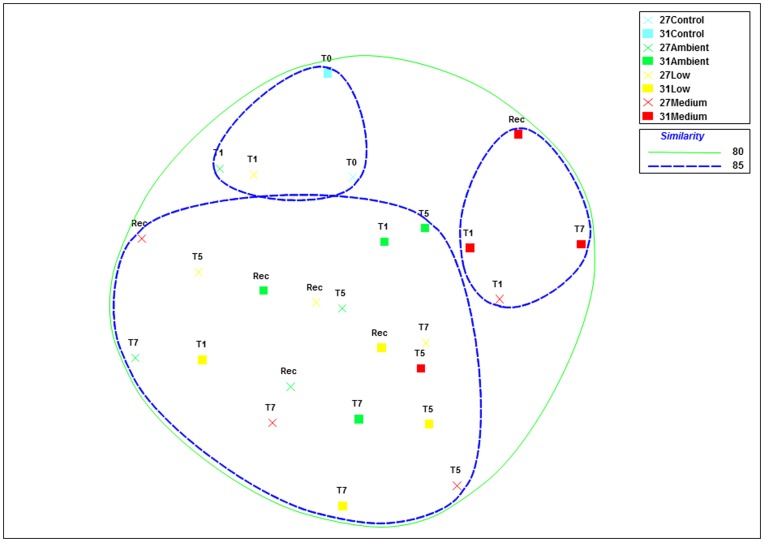
MDS (multidimensional scaling) ordination of bacterial communities amongst samples as derived from 16S rRNA-DGGE profiles. Banding patterns were transformed into a presence (scored as 1)/absence (scored as 0) matrix. MDS plots were generated using distance matrices to represent the relative distance between individual samples. Colours represent nutrient treatments: blue = control, green = ambient, yellow = low and red = medium nutrient level exposure. Crosses represent 27°C-exposed sponges and squares represent 31°C-exposed sponges. T = day of sampling and REC = recovery period (ambient nutrient levels and seawater temperatures). The final stress value of the plot was 0.21. Cluster analyses for similarity are indicated by colored contours at 80–85% similarity.

Nutrient exposures were selected using inshore data reported from the northern GBR and from previous research which demonstrated that our chosen exposure levels can cause stress in corals (Tables 1 and 2) [36], [45], [47], [89]. Our highest nutrient treatment levels resulted in 9-fold, 7.5-fold, 7-fold and 2.1-fold enrichments of ammonium, phosphate, nitrite and nitrate respectively (recorded from ambient exposures over the duration of the experiment (Table S1b)). *R. odorabile* clones exposed to all nutrient and temperature treatments remained visibly healthy throughout the duration of the experiment (Figure S1). 16S rRNA gene-targeting DGGE was used to screen all samples for changes in dominant members of the bacterial community. Most DGGE bands were highly conserved across all sponge clones, time periods, and nutrient/temperature exposures, revealing that microbial community composition is largely unaffected by the combined effects of elevated nutrients and sub-lethal temperature. A multidimensional scaling **(**MDS) plot combined with cluster analysis of DGGE profiles (Figure 1) revealed no clustering according to experimental treatment, with at least 80% similarity detected between all samples. As sponge health and the dominant microbial community did not appear to change at the highest nutrient/temperature treatment, only a subset of samples (day 0 control and day 7 samples from all treatments) were screened by 454 pyrotag sequencing to assess whether changes occur in the rare microbial biosphere.

### Nutrient and Sub-lethal Thermal Stress Effects on the Bacterial Biosphere

After noise reduction and quality filtering (see Material and Methods) a total of 35757 sequences was obtained from a total of 15 samples, with mean = 2384 (±346 (1SD)) sequences per sample (Table S2). For each sample, less than 0.05% (±0.07 (1SD)) of reads were taxonomically unassigned at phylum level. In total, 1743 OTUs (97% sequence similarity, Table S2) were identified, affiliated with 19 bacterial phyla. Rarefaction curves (Figure S2) indicated that, while further sequencing would have yielded a greater number of OTUs, diversity coverage was high with most curves approaching asymptotes. With the exception of sample 727LC, the observed number of OTUs was similar across all samples between 210–339 OTUs (Table S2).

**Figure 2:**
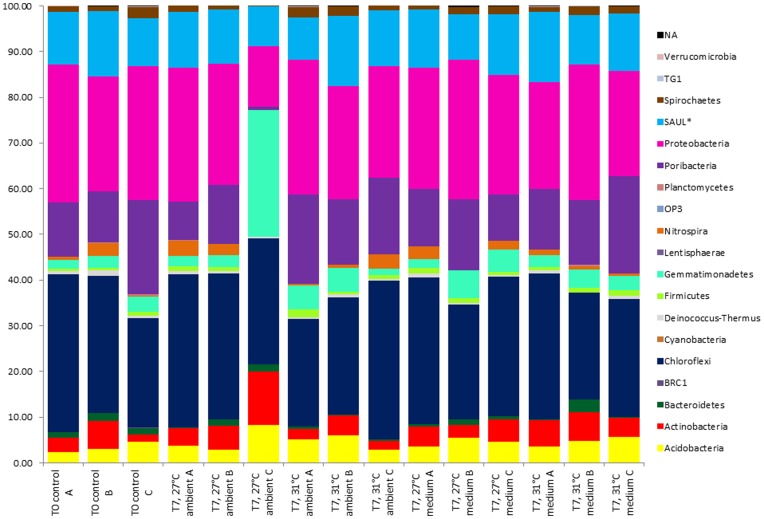
Distribution of 454 amplicon reads per phylum across nutrient/temperature treatments. The number of reads per phylum is calculated as a percentage of the total reads in each sample. Samples are grouped in replicates (A, B, C), according to nutrient (ambient or medium)/temperature exposure (27 or 31°C) and ordered by sampling date (Days 0 and 7). *SAUL (sponge-associated unidentified lineage [87]). NA (not assigned).

**Figure 3:**
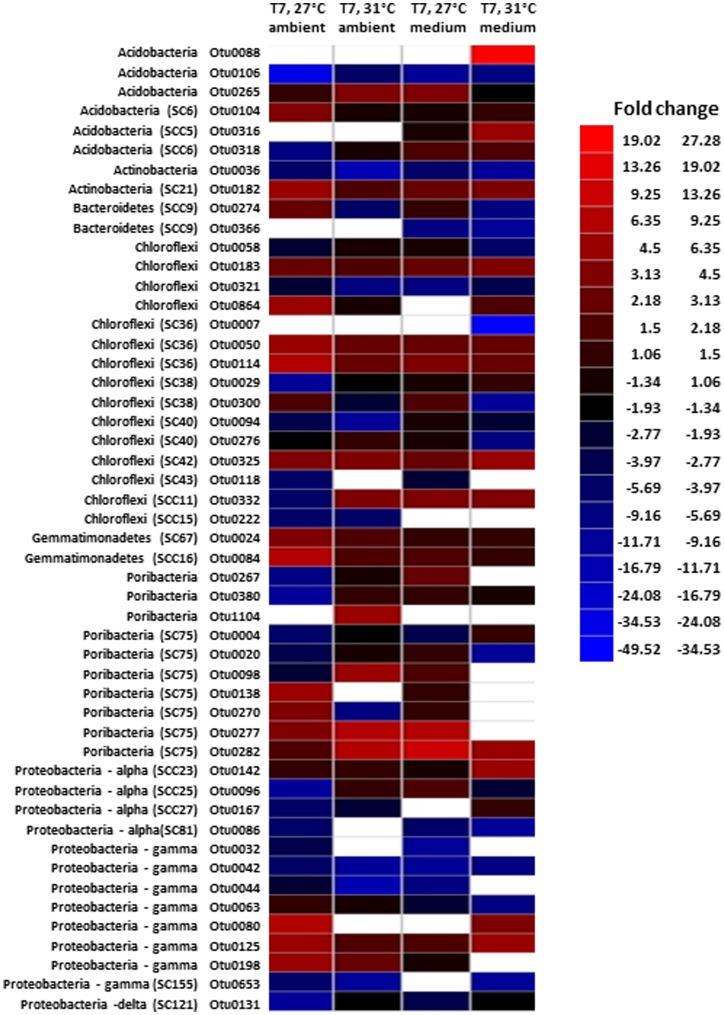
Heatmap of the 50 OTUs (from Day 7) with the largest negative or positive fold-change from Day 0 controls. Averages of three replicates were used for fold-change calculations. Treatment (ambient or medium nutrient exposure/27°C or 31°C) values were divided by the relevant control values (Day 0). Fold-changes were Log (base 2)-transformed, so that positive (increases in relative abundance) and negative values (decreases) were weighted equally. Key: Black squares representing no change from control value, red scale representing positive fold changes, blue scale representing negative fold changes and white squares indicating no data in control or treatment.

Phylum-level composition of bacterial communities was highly conserved across all samples (Figure 2). The dominant phyla were *Chloroflexi, Proteobacteria*, “*Poribacteria”* and Sponge-Associated Unidentified Lineage (SAUL), representing on average 29, 26, 14, and 12% of sequences, respectively, across all samples. Within the *Proteobacteria* most sequences were either *Delta-* or *Gammaproteobacteria* (13 and 10% respectively of total bacterial sequences). Phyla that were less abundant but found in all samples included the *Acidobacteria* (5%), *Actinobacteria* (5%) and *Gemmatimonadetes* (5%). Replicates from each treatment exhibited highly conserved bacterial community compositions, except for sample 727LC, which harbored the majority of phyla detected in the other samples but in markedly different proportions. Notably, the *Gemmatimonadetes* increased in proportion (27%) and there were very few “*Poribacteria”* (0.71%) in sample 727LC. Comparison of bacterial community composition of individual samples was tested, at the OTU level, using a non-metric multidimensional scaling (NMDS) plot, which showed all samples clustered tightly together with only sample 727LC separating on the ordination (Figure S3, stress = 0.09, R^2^ = 0.97). Unweighted Unifrac [90] analysis also revealed there were no significant differences between treatments at the OTU level. The 50 OTUs with the largest number of reads as determined by pyrosequencing were calculated (Figure S4) and together represented a range between 66–84% of total reads across all samples. These results support the phylum-level composition data, with most of the top 50 OTUs affiliated with the *Chloroflexi*, *Proteobacteria* and “*Poribacteria”*, and abundances of individual OTUs were highly similar across all treatments. The primary differences occurred in replicate 727LC, which had a very high abundance of reads (34% in total) assigned to OTU0014 (*Gemmatimonadetes*), OTU0024 (*Gemmatimonadetes*) and OTU0084 (*Chloroflexi*). As no substantial shifts in bacterial community composition at phylum or OTU level were observed in any other sample, values were calculated for the 50 OTUs with the largest (negative or positive) fold change, per nutrient/temperature exposure relative to Day 0 controls (Figure 3). Most of the top 50 OTU fold changes occurred in the *Chloroflexi* (30% of the top 50 fold-changes), *Proteobacteria* (26%) and “*Poribacteria”* (20%). Some trends were observed across all samples, for example OTUs belonging to the *Gemmatimonadetes* increasing in abundance and *Deltaproteobacteria* OTUs decreasing in abundance across all samples. Two OTUs (0088 and 0316) in the *Actinobacteria* only showed positive fold changes (abundance increases) in sponge clones exposed to the high nutrient treatments. BLAST searches revealed that these OTUs were closely affiliated to other sponge symbionts (data not shown). However, overall no major shifts in OTU fold change occurred due to elevated nutrient levels (with/without temperature treatment).

The proportion of reads assigned to one of the previously described SCs/SCCs [22] was highly similar across all nutrient and temperature exposures (Figure S5a) (63%–83%). Except for replicate sample 727LC, most SC clusters belonged to the *Chloroflexi, Proteobacteria* and “*Poribacteria”* (Figure S5b) and the majority of SCC were assigned to the *Chloroflexi, Gemmatimonadetes* and *Alphaproteobacteria* (Figure S5c). Of the top 50 OTUs with the largest (negative or positive) fold-change, 64% fell into SC/SCC clusters, mostly within these same phyla.

### Nutrient and Sub-lethal Thermal Stress Effects on Archaeal Community Structure

**Figure 4:**
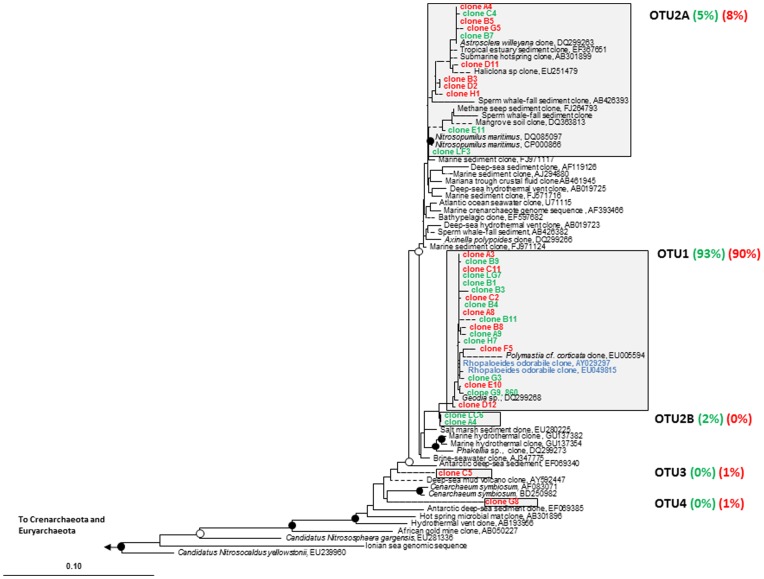
Maximum likelihood phylogenetic tree from analysis of 16S rRNA gene sequences of archaeal clone libraries. The tree is constructed based on long (≥1200 bp) sequences only; shorter sequences were added using the parsimony interactive tool in ARB and are indicated by dashed lines. Filled circles indicate bootstrap support (maximum parsimony, with 1000 resamplings) of ≥90%, and open circles represent ≥75% support. Bar, 10% sequence divergence. Sequences from medium nutrient clone library are in red and ambient library in green. Blue sequences represent archaea previously reported from healthy *R. odorabile* (Webster et al., 2001). The proportion of each OTU from treatment libraries (red or green) are in parentheses.

**Figure 5:**
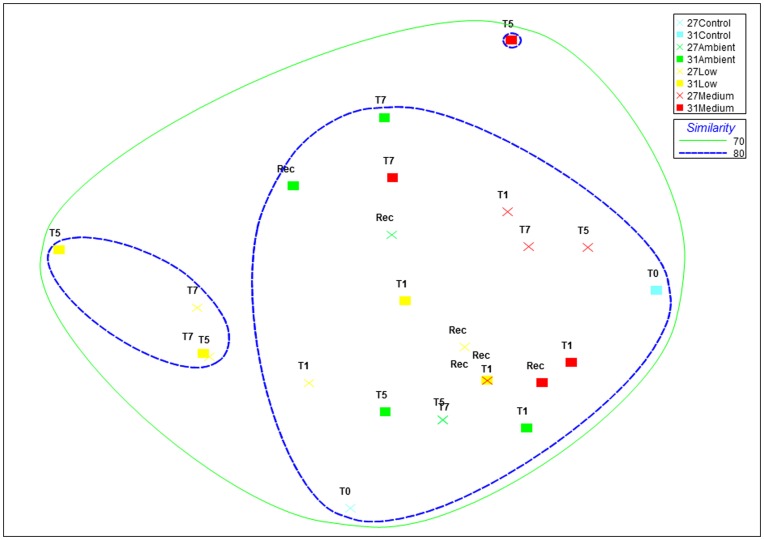
MDS (multidimensional scaling) ordination of *R. odorabile* -derived archaeal *amoA* genes as derived from DGGE profiles. Banding patterns were transformed into a presence (scored as 1)/absence (scored as 0) matrix. MDS plots were generated using distance matrices to represent the relative distance between individual samples. Colours represent nutrient treatments: blue = control, green = ambient, yellow = low and red = medium nutrient level exposure. Crosses represent 27°C-exposed sponges and squares represent 31°C-exposed sponges. T = day of sampling and REC = recovery period (ambient nutrient levels and seawater temperatures). The final stress value of the plot was 0.18. Cluster analyses for similarity are indicated by colored contours at 70–80% similarity.

To determine the diversity of archaeal sequences, two 16S rRNA gene libraries were created, one from sponge clones kept in ambient nutrient conditions and a second from sponges exposed to the medium nutrient treatment. A total of 171 clones were screened by ARDRA analysis. ARDRA patterns revealed five unique OTUs (Figure 4), all associated with *Thaumarchaeota* (previously called *Crenarchaeota* Marine Group I). OTU1 and OTU2 were present in both ambient and medium nutrient treatment libraries, with OTU1 dominating both libraries (ambient = 93% and medium = 90%) and clustering with archaea previously reported from healthy *R. odorabile*. The ARDRA pattern that gave rise to OTU2 produced two distinct clusters (OTU2A and OTU2B), with OTU2B only present in the ambient nutrient library at 2%. The least common ARDRA patterns gave rise to OTUs 3 and 4, which were only present in the medium nutrient clone libraries at 1% each, with OTU4 related to *Cenarchaeum symbiosum* [28]. To determine whether nutrient enrichment altered potential functionality we also targeted the *amoA* gene in ammonia-oxidizing archaea (AOA) using DGGE. Variations in DGGE banding patterns were not correlated with any time points, nutrient or temperature treatments. MDS plots (Figure 5) clustered all samples together with at least 70% similarity, confirming that differences in AOA diversity are not related to nutrient or temperature stress.

### Nutrient and Sub-lethal Thermal Stress Effects on Community Structure of Microbial Eukaryotes

**Figure 6:**
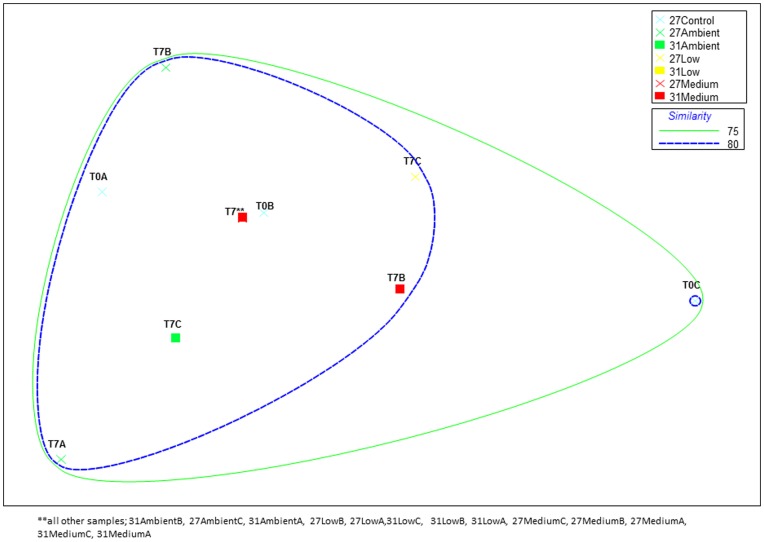
MDS (multidimensional scaling) ordination of the eukaryotic microbial communities of *R. odorabile* as derived from DGGE profiles. Banding patterns were transformed into a presence (scored as 1)/absence (scored as 0) matrix. MDS plots were generated using distance matrices to represent the relative distance between individual samples. Colours represent nutrient treatments: blue = control, green = ambient, yellow = low and red = medium nutrient level exposure. Crosses represent 27°C-exposed sponges, squares represent 31°C-exposed sponges and T = day of sampling. The final stress value of the plot was 0.03. Cluster analyses for similarity are indicated by colored contours at 75–80% similarity.

The majority of eukaryote-specific DGGE banding patterns were highly conserved regardless of nutrient/temperature treatment. MDS plots of DGGE banding pattern profiles confirmed eukaryotic community composition was highly similar across all treatments (Figure 6) and all samples clustered together with at least 75% similarity.

## Discussion

*R. odorabile* clones exposed to the combined effects of elevated nutrient levels and seawater temperature appeared visually similar to those maintained under ambient seawater conditions. The microbial communities of *R. odorabile* were not significantly affected by these environmental stressors, indicating that this sponge species is capable of withstanding short-term exposure to elevated nutrient concentrations and sub-lethal temperatures. *R. odorabile* is found throughout the GBR, occurring on inner-, mid- and outer-reef locations [91], [92]. The broad distribution of *R. odorabile* throughout the GBR exposes this species to a well-defined water quality gradient, with inner reef sponges experiencing higher nutrient loads, particularly during flood events, compared to mid and outer reef sponges [6], [93]. While reproductive output has been reported to decrease in female *R. odorabile* from inner reefs compared to outer reefs, these changes could not be directly linked to elevated nutrients or water turbidity [94]. Many cases have shown that microbial communities are sensitive to environmental perturbation [48], [95]. The evidence presented here, however, suggests that microbial communities within *R. odorabile* can resist these nutrient perturbations, even at temperatures of 31°C.

Eutrophication and poor water quality are major concerns for reef ecosystems globally. In addition to local factors, coral reefs are also faced with global stressors including elevated sea surface temperatures and ocean acidification [96], [97]. Despite this, the interacting effects of multiple environmental stressors on marine invertebrates are seldom investigated. Ambient levels of nitrogen and phosphorus recorded over the duration of the experiment were higher than those for nearby reefs, which is potentially due to input from the Orpheus Island Research Station. Sponges were exposed to nutrient concentrations 9-fold, 7.5-fold, 7-fold and 2.1-fold (ammonium, phosphate, nitrite and nitrate concentrations, respectively) above ambient yet showed no adverse health effects or changes in symbiosis. These results are further supported by a recent study that found nutrient enrichment does not affect the sponge *Aplysina cauliformis* or its symbiont community [98]. Healthy and Aplysina Red Band Syndrome (ARBS)-affected *A. cauliformis* were exposed to nutrient-enriched conditions (up to 4.8-fold and 2.1-fold increases of nitrate and phosphate respectively, from ambient levels over 7 days). A combination of terminal restriction fragment length polymorphism (T-RFLP), histology and chlorophyll fluorescence measurements [98] revealed no change in the bacterial communities of healthy sponges, nor an enhanced rate of disease progression in ARBS-affected sponges. However, nutrient enrichment levels similar to those in this experiment have been shown to exacerbate the onset and severity of coral diseases, including Black Band Disease [42], aspergillosis and Yellow Band Disease [36]. Although the mechanisms are unknown, this may be due to an enhancement of microbial growth rates [45] and/or increased pathogen virulence [36], [37].

The microbial community of *R. odorabile* analyzed by 454 pyrotag sequencing was highly conserved for the duration of the experiment at both phylum and OTU levels. Consistent with other sponge amplicon pyrosequencing studies [19], [20], [87], [99], communities were dominated by *Chloroflexi, Proteobacteria, “Poribacteria”* and SAUL at both the phylum and OTU level. Whilst the abundance data and fold change data for the majority of OTUs showed little correlation with particular nutrient/temperature treatments, replicate C at time 7 days, seawater temperature 27°C and ambient concentrations of nutrients (727LC) was an exception. In 727LC the differences were attributed to two *Gemmatimonadetes* OTUs and one OTU within the *Chloroflexi*. Given the high similarity of all other replicates and that this clone appeared visibly healthy, it is possible that this anomaly was a consequence of associated infauna being inadvertently sequenced with the sponge tissue. Sponge-specific clusters (SC) and sponge-coral-specific clusters (SCC) [17], [21], [22] are monophyletic clusters of 16S rRNA sequences found only in sponges (or sponges and corals) and not the surrounding environment such as seawater or sediments. We saw neither a significant increase nor decrease in the proportion of reads assigned to SCs or SCCs, with approximately 70% of all reads assigned to these clusters per sample across all treatments. Even though the roles of SCs/SCCs are still largely uncharacterized, it is predicted that their loss would be detrimental to the health and survival of the host sponge [100].

Nutrient enrichment and sub-lethal temperature stress did not alter the symbiotic archaeal associations within *R. odorabile*. Archaeal sequences were affiliated with *Thaumarchaeota*, which is consistent with archaeal sequences previously found in this species and in other sponge studies [101]–[103]. To address changes in community composition and potential functionality of sponge-associated archaea due to elevated nutrients and temperature, we also screened samples for changes in the *amoA* gene. In AOA and AOB the AmoA enzyme catalyses aerobic oxidation of ammonia to nitrite (the first step of nitrification). Analysis by qPCR in both the marine and terrestrial environment has suggested that AOA outnumber AOB [76], [104], [105] and this is also the case in at least some cold water marine sponges [29]. In this study the highest level of ammonium that sponges were exposed to was 9-fold higher than ambient levels. However, no shifts in the composition of AOA could be correlated with nutrient or temperature treatment, indicating that the diversity of AOA is stable under multiple environmental stressors.

Marine eukaryotic microbial communities mainly consist of algae, protozoa and marine fungi which play important roles in microbial food webs and in nutrient cycling [106], [107]. Within marine sponges, diatoms, dinoflagellates and fungi are known to live symbiotically although their functional roles within the sponge remain unclear [17], [108]. The effects of eutrophication on free-living eukaryotic microbial communities are widely reported: as the availability of nitrogen and phosphorus increases, primary production by algae is stimulated and the structures of phytoplankton communities change [109]–[112]. Ultimately these changes lead to increased turbidity in the water column coupled with oxygen depletion, which can greatly affect benthic communities [109]. Whilst the effect of nutrient amendment on host-associated microbial communities is less understood, nutrient enrichment studies with corals have shown increases in zooxanthellae abundance and indicate that these cells have preferential access to available CO_2_ which is then used for photosynthesis. Within the sponge *Cymbastela concentrica*, nutrient enrichment had no effect on symbiotic micro-algal growth as detected via chlorophyll concentration and sponge growth rate [113]. In the current study, the conserved eukaryotic community in *R. odorabile* across all nutrient and temperature treatments further highlights the stability of microbial associations within this sponge species. Based on eutrophication studies with free-living systems, one possible scenario resulting from nutrient elevation is an increase in the relative abundance of some species (particularly photosynthetic organisms). However, further exploration is required to investigate this.

Here we exposed sponges to seawater temperatures of 31°C which, based on Intergovernmental Panel on Climate Change (IPCC) scenarios, will occur before 2100 [114]. Previous studies assessing the response of *R. odorabile* to thermal stress have indicated sub-lethal effects at 31°C, including activation of the heat shock protein system [68] and a significant decrease in flow rate, filtration efficiency and choanocyte chamber density [115]. Whilst the bacterial community of *R. odorabile* is highly stable at 31°C, higher seawater temperatures cause a shift in the symbiont community which is concomitant with host tissue necrosis and mortality after four days at 32°C or three days at 33°C [67], [69]. Anthropogenic stressors such as water pollution have been shown to negatively interact with elevated seawater temperature, reducing coral larval metamorphosis [59] and increasing the persistence of the coral pathogen *Serratia marcescens* [41]. Research on coral bleaching thresholds also identified higher temperature sensitivity after exposure to increased DIN concentrations [116] with a recent study confirming that increased DIN, combined with limited phosphate concentrations, increases the susceptibility of corals to temperature- and light-induced bleaching. This is thought to occur due to an imbalanced DIN supply causing phosphate starvation of the symbiotic zooxanthellae [61]. In contrast, sub-lethal thermal stress in the current study did not appear to increase the susceptibility of *R. odorabile* to elevated nutrients.

Our study generated nutrient enrichment levels that are known to occur during major flood plume events and which have a destabilizing effect on the host-symbiont relationship in corals. The different response of sponges and corals to nutrient treatment may relate to the short timescale of exposure used in our study. However, the time scale of this study reflects real-time dispersal rates of elevated nutrients in the GBR environment. Many studies have reported the detrimental impacts of various water quality parameters on sponges and corals directly [36], [40], [42], [89], [94], [117]–[120]. In contrast, we report for the first time the effects of multiple environmental stressors on the important partnerships that reef invertebrates form with symbiotic microbes. We detected no changes in the bacterial, eukaryotic or archaeal community from any of the nutrient and/or temperature treatments, indicating that *R. odorabile* will be able to withstand nutrient pulses associated with flood plume events that are becoming more frequent in occurrence and severity [2], [13], [14], [93]. By assessing multiple stressors in combination, this study provides a first step for environmentally relevant sponge stress assessments which will enhance management strategies for GBR sponge populations.

## Supporting Information

Figure S1 *R. odorabile* clones at the end of the recovery phase (12 days). Clones shown are (A) T = 12, ambient nutrient exposure and 31°C (B) T = 12, high nutrient exposure and 31°C. Large boxes show the entire sponge clone, smaller boxes show internal mesohyl tissue, from each respective sponge clone.
(TIF)

Figure S2 Bacterial diversity of all sponge samples amplified with 454 amplicon pyrosequencing. Rarefaction curves are based on OTUs at 97% sequence similarity. Calculations were performed in Mothur (Schloss et al., 2009)
(TIF)

Figure S3 Comparison of bacterial community composition of individual samples was tested, at the OTU level, using an nMDS plot. Lowest stress = 0.0904, R^2^ = 0.9711. Calculations were performed in Mothur (Schloss et al., 2009). Unweighted Unifrac analysis also revealed there were no significant differences between time points at the OTU level.
(TIF)

Figure S4 The relative abundance of the 50 most abundant OTUs (according to the sum of relative abundance across all samples). Samples are clustered according to phylogenetic affiliation. Scale is percentage of reads in each OTU (white = 0%). C = control with replicate A, B or C. A = ambient nutrient treatment with replicate A, B or C. M = medium nutrient treatment with replicate A, B or C
(TIF)

Figure S5 (a) The proportion of reads that were assigned to an SC or SCC per sample (b) The proportion of reads that were assigned to an SC per bacterial phylum and (c) The proportion of reads that were assigned to an SCC per bacterial phylum. The number of reads per phylum (b and c) is calculated as a percentage of the total reads that were assigned to a SC/SCC in each sample.
(TIF)

Table S1 **A.** Average nutrient parameters measured in the experiment for ambient, low, medium and recovery nutrient exposure levels (n = 3 for each nutrient level). **B.** Average fold change in nutrient concentrations measured (for low, medium and recovery) during the course of the experiment from ambient values (n = 3 for each nutrient level).
(TIF)

Table S2 Overview of the total number of sequence reads obtained per sample and the total numbers of OTUs obtained per sample are shown (after noise removal, quality filtering etc.) OTU information is reported at 97% similarity
(TIF)
