# Supplementary figures and images for: Sponge-Microbe Associations Survive High Nutrients and Temperatures
Source: PLoS One. 2012 Dec 20;7(12):e52220. doi: 10.1371/journal.pone.0052220 (PMC3527390; doi:10.1371/journal.pone.0052220)

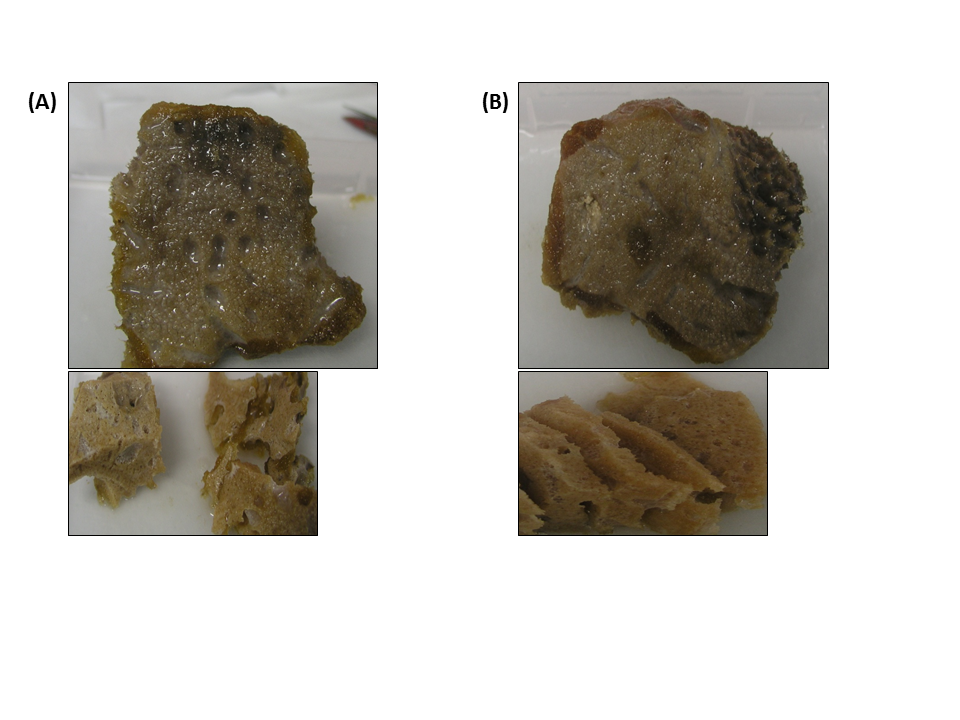

Supplement: Figure S1 — R. odorabile clones at the end of the recovery phase (12 days). Clones shown are (A) T = 12, ambient nutrient exposure and 31°C (B) T = 12, high nutrient exposure and 31°C. Large boxes show the entire sponge clone, smaller boxes show internal mesohyl tissue, from each respective sponge clone. (TIF) [file pone.0052220.s001.tif]

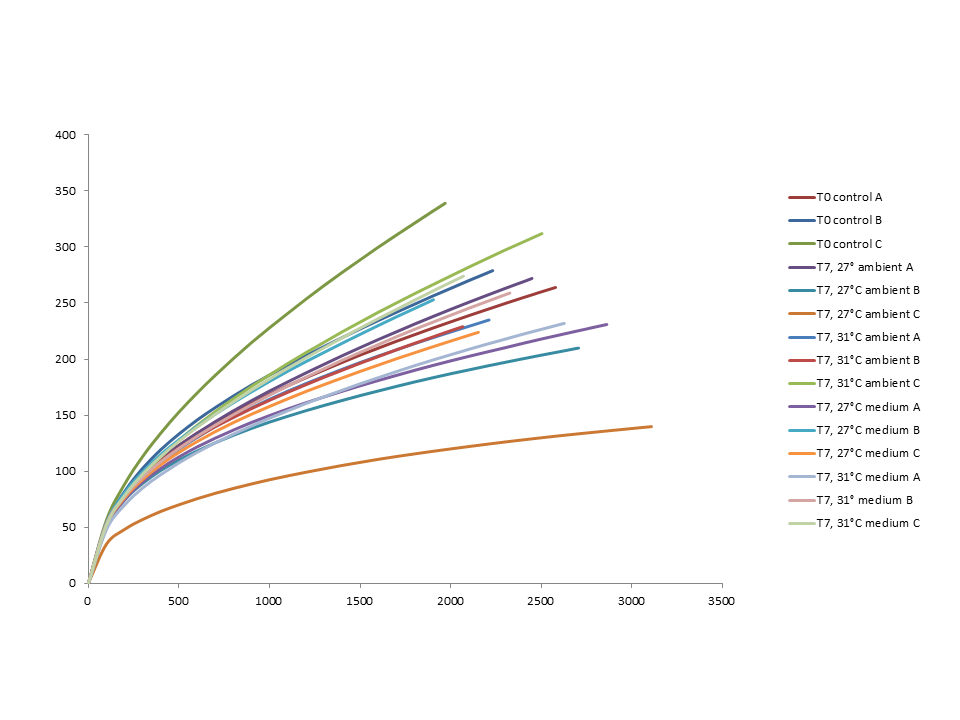

Supplement: Figure S2 — Bacterial diversity of all sponge samples amplified with 454 amplicon pyrosequencing. Rarefaction curves are based on OTUs at 97% sequence similarity. Calculations were performed in Mothur (Schloss et al., 2009) (TIF) [file pone.0052220.s002.tif]

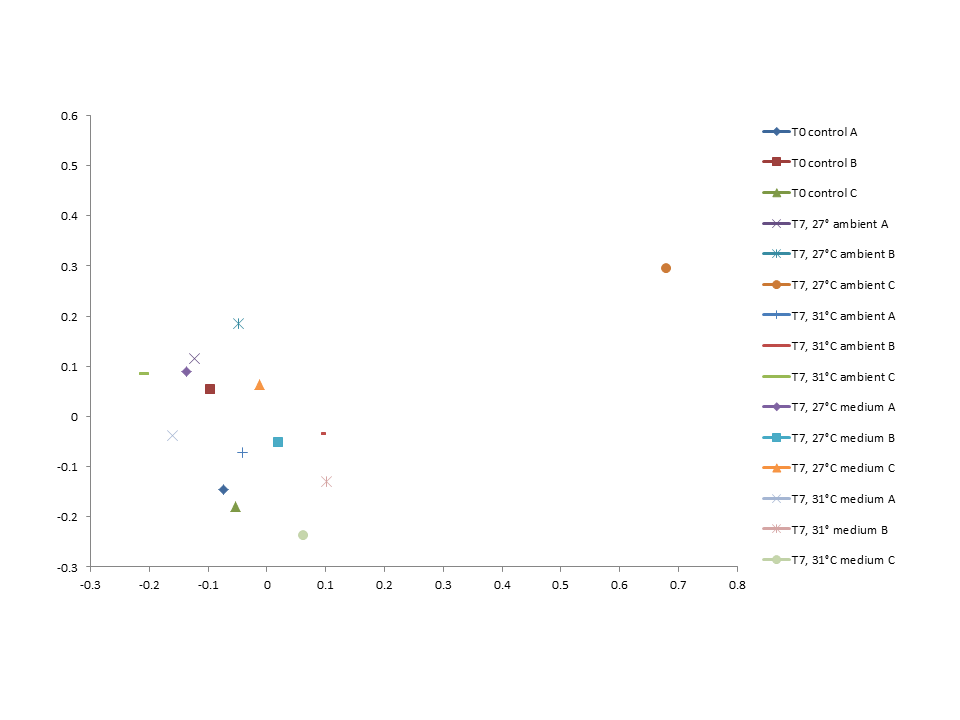

Supplement: Figure S3 — Comparison of bacterial community composition of individual samples was tested, at the OTU level, using an nMDS plot. Lowest stress = 0.0904, R2 = 0.9711. Calculations were performed in Mothur (Schloss et al., 2009). Unweighted Unifrac analysis also revealed there were no significant differences between time points at the OTU level. (TIF) [file pone.0052220.s003.tif]

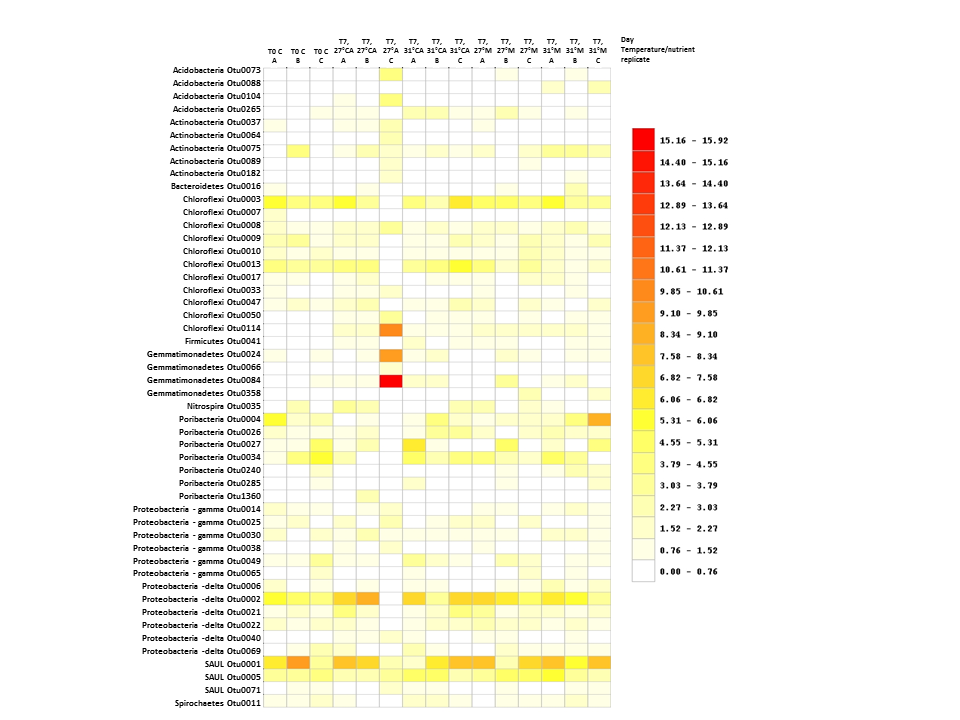

Supplement: Figure S4 — The relative abundance of the 50 most abundant OTUs (according to the sum of relative abundance across all samples). Samples are clustered according to phylogenetic affiliation. Scale is percentage of reads in each OTU (white = 0%). C = control with replicate A, B or C. A = ambient nutrient treatment with replicate A, B or C. M = medium nutrient treatment with replicate A, B or C (TIF) [file pone.0052220.s004.tif]

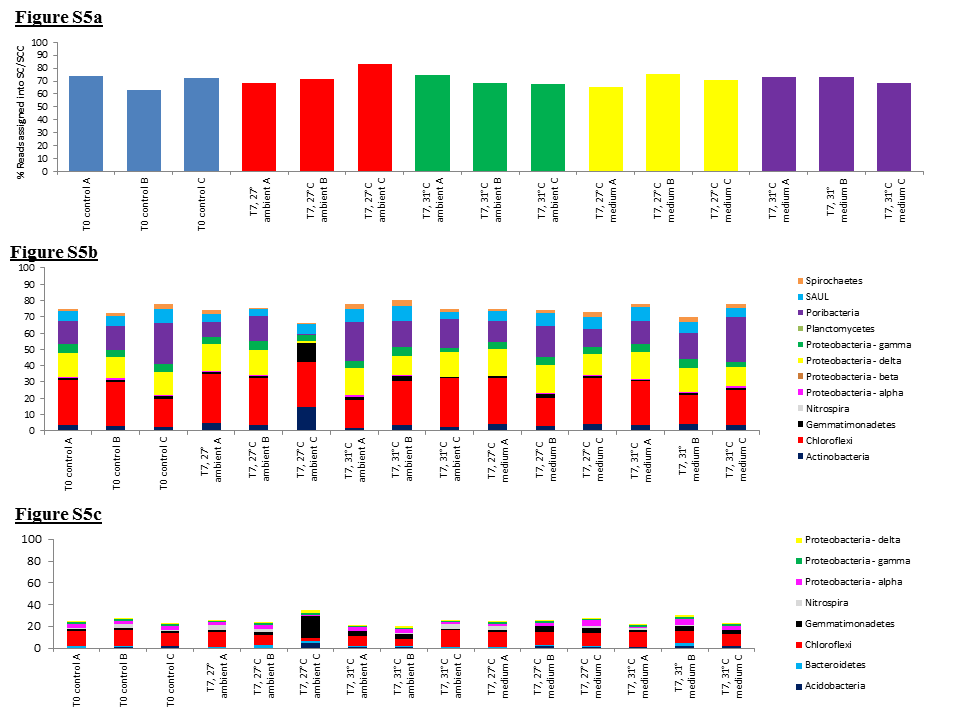

Supplement: Figure S5 — (a) The proportion of reads that were assigned to an SC or SCC per sample (b) The proportion of reads that were assigned to an SC per bacterial phylum and (c) The proportion of reads that were assigned to an SCC per bacterial phylum. The number of reads per phylum (b and c) is calculated as a percentage of the total reads that were assigned to a SC/SCC in each sample. (TIF) [file pone.0052220.s005.tif]
